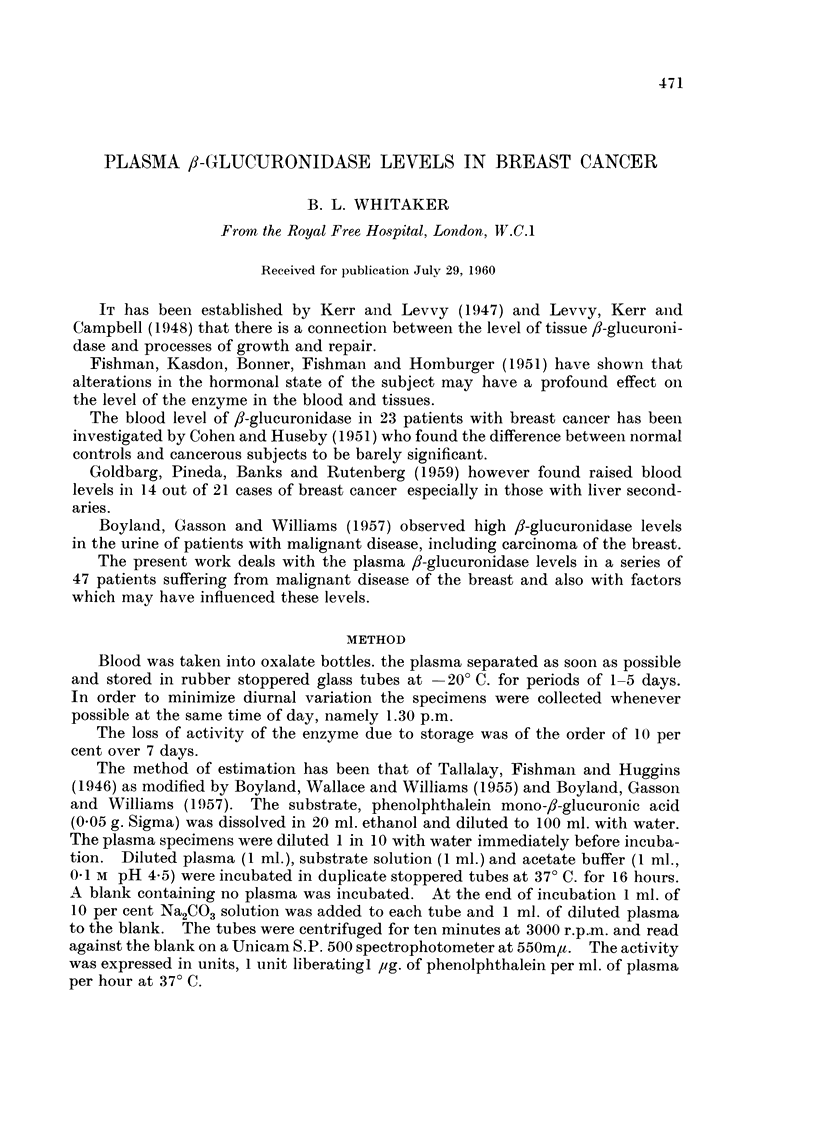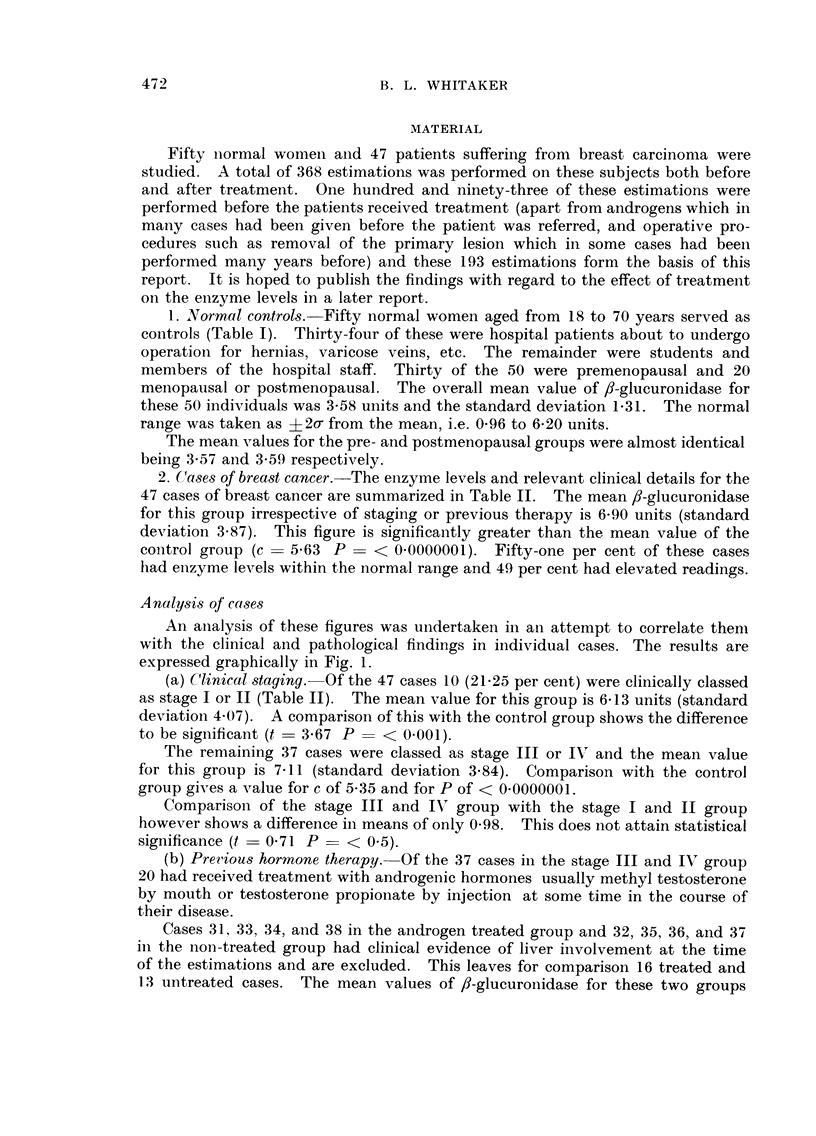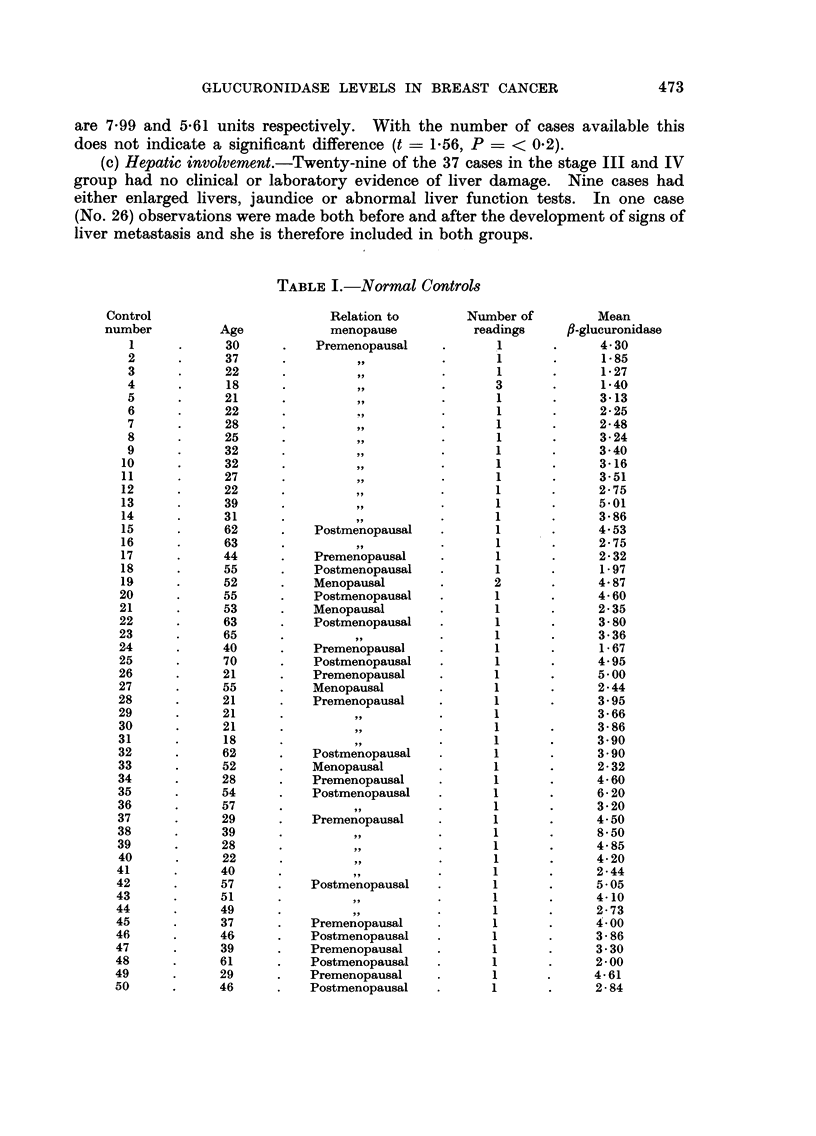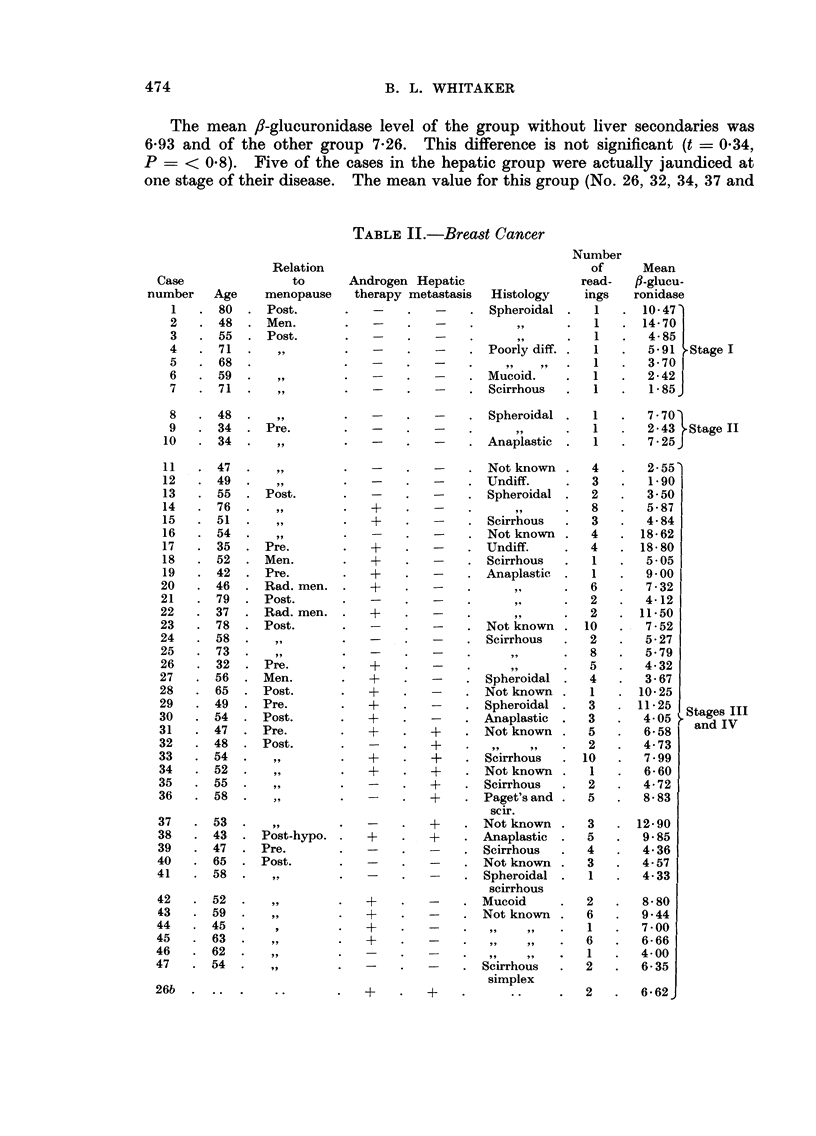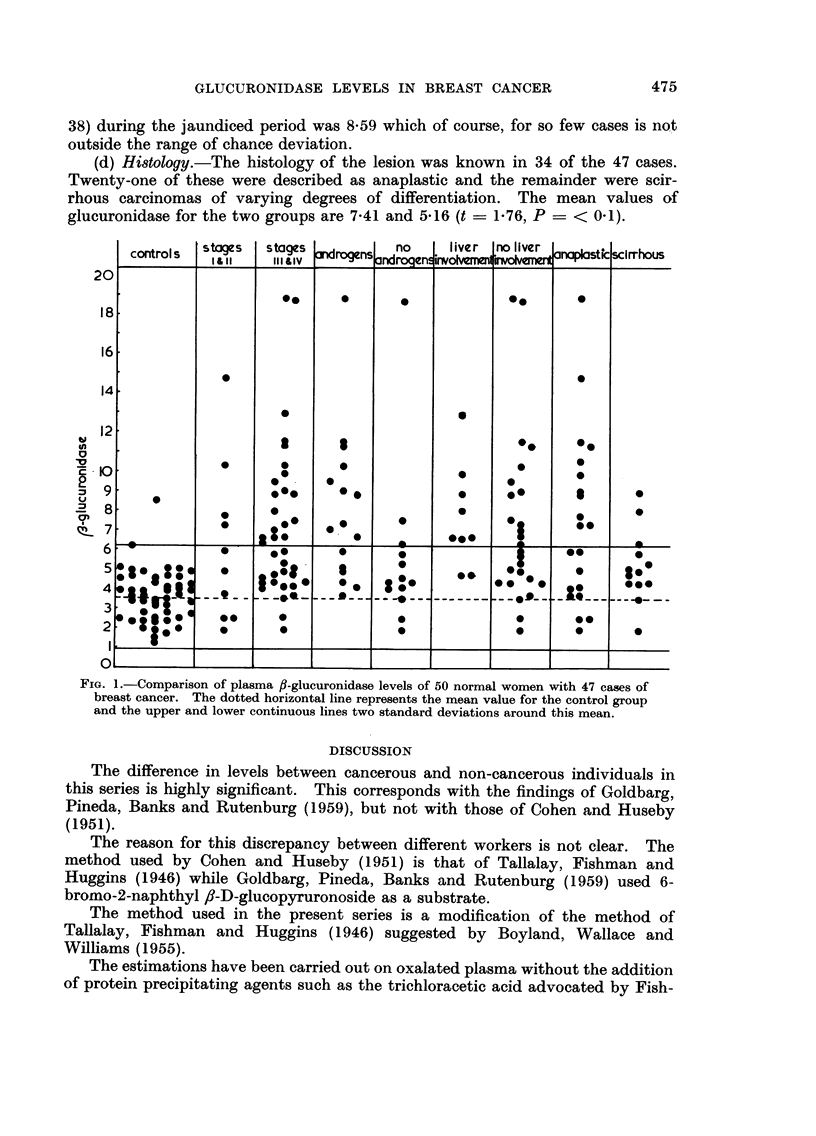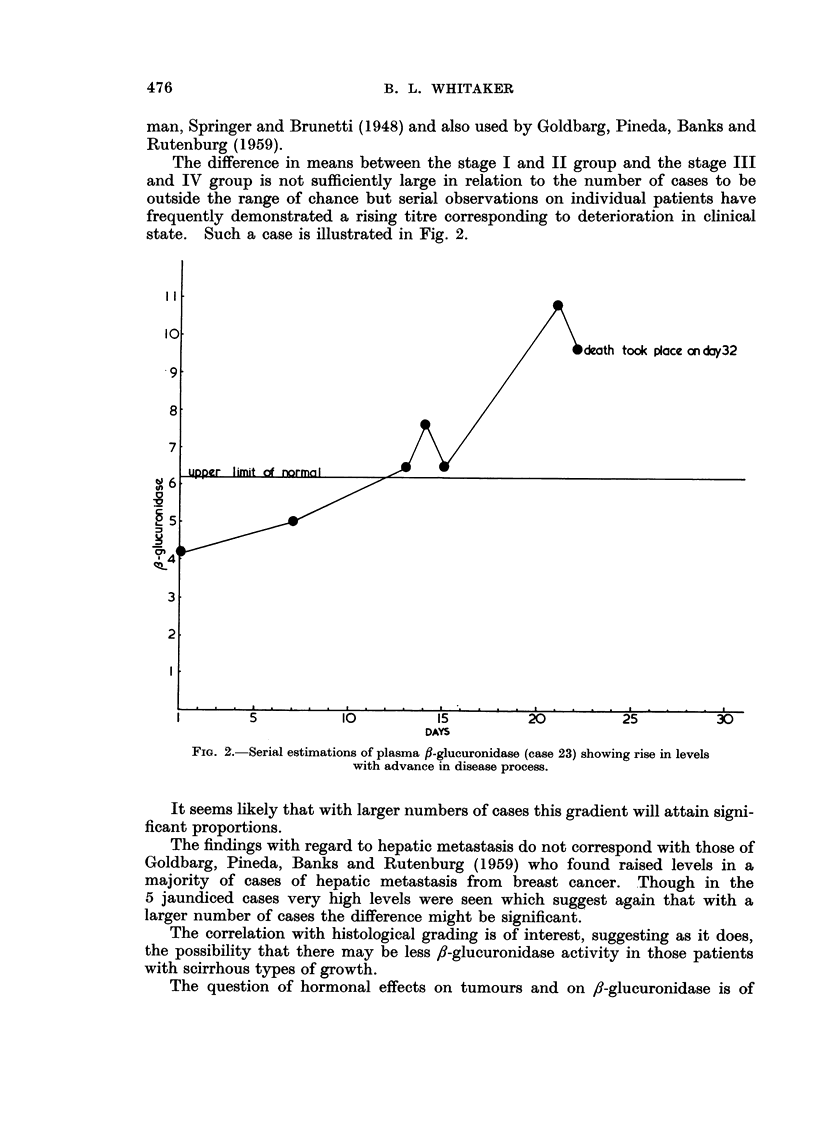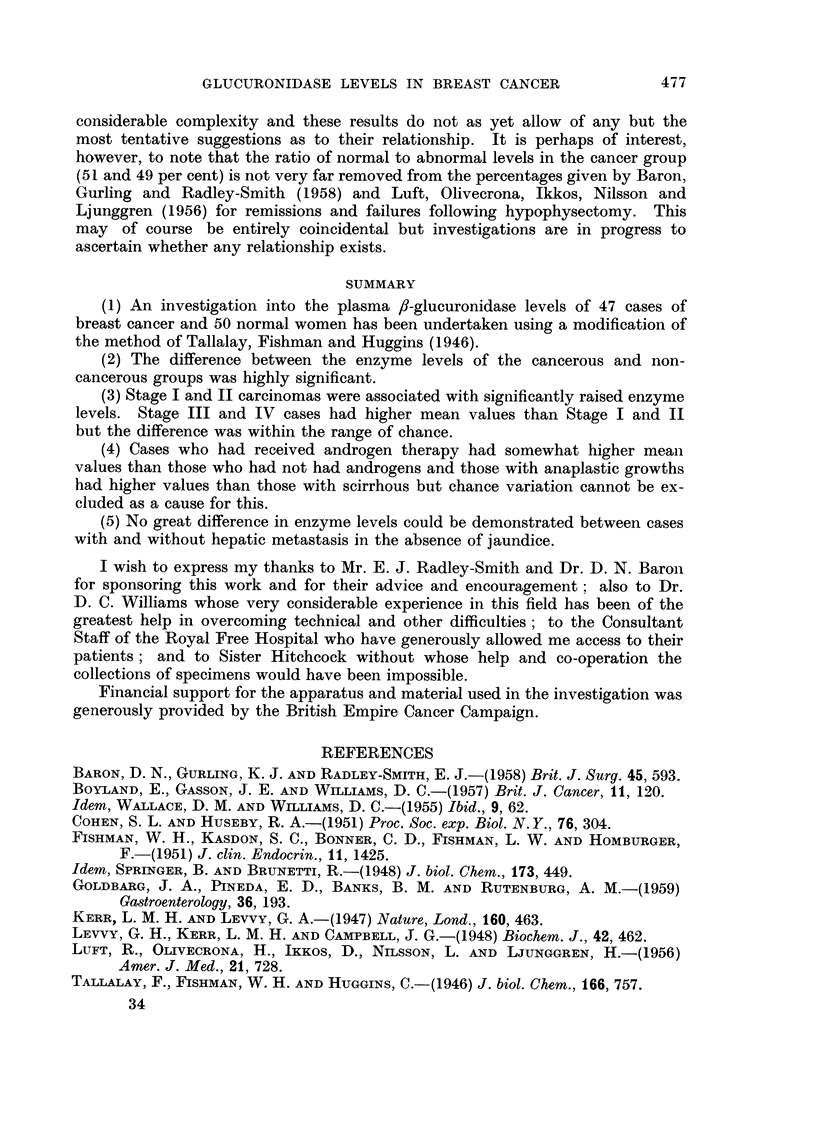# Plasma β-Glucuronidase Levels in Breast Cancer

**DOI:** 10.1038/bjc.1960.51

**Published:** 1960-09

**Authors:** B. L. Whitaker


### PLASMA β-GLUCURONIDASE LEVELS IN BREAST CANCER

B. L. WHITAKER

*From the Royal Free Hospital, London, W.C.*1

Received for publication July 29, 1960

It has been established by Kerr and Levvy (1947) and Levvy, Kerr and Campbell (1948) that there is a connection between the level of tissue β-glucuronidase and processes of growth and repair.

Fishman, Kasdon, Bonner, Fishman and Homburger (1951) have shown that alterations in the hormonal state of the subject may have a profound effect on the level of the enzyme in the blood and tissues.

The blood level of β-glucuronidase in 23 patients with breast cancer has been investigated by Cohen and Huseby (1951) who found the difference between normal controls and cancerous subjects to be barely significant.

Goldbarg, Pineda, Banks and Rutenberg (1959) however found raised blood levels in 14 out of 21 cases of breast cancer especially in those with liver secondaries.

Boyland, Gasson and Williams (1957) observed high β-glucuronidase levels in the urine of patients with malignant disease, including carcinoma of the breast.

The present work deals with the plasma β-glucuronidase levels in a series of 47 patients suffering from malignant disease of the breast and also with factors which may have influenced these levels.

### METHOD

Blood was taken into oxalate bottles. the plasma separated as soon as possible and stored in rubber stoppered glass tubes at $-20°$ C. for periods of 1–5 days. In order to minimize diurnal variation the specimens were collected whenever possible at the same time of day, namely 1.30 p.m.

The loss of activity of the enzyme due to storage was of the order of 10 per cent over 7 days.

The method of estimation has been that of Tallalay, Fishman and Huggins (1946) as modified by Boyland, Wallace and Williams (1955) and Boyland, Gasson and Williams (1957). The substrate, phenolphthalein mono-β-glucuronic acid (0·05 g. Sigma) was dissolved in 20 ml. ethanol and diluted to 100 ml. with water. The plasma specimens were diluted 1 in 10 with water immediately before incubation. Diluted plasma (1 ml.), substrate solution (1 ml.) and acetate buffer (1 ml., 0·1 M pH 4·5) were incubated in duplicate stoppered tubes at 37° C. for 16 hours. A blank containing no plasma was incubated. At the end of incubation 1 ml. of 10 per cent $Na_2CO_3$ solution was added to each tube and 1 ml. of diluted plasma to the blank. The tubes were centrifuged for ten minutes at 3000 r.p.m. and read against the blank on a Unicam S.P. 500 spectrophotometer at 550m$\mu$. The activity was expressed in units, 1 unit liberating1 $\mu$g. of phenolphthalein per ml. of plasma per hour at 37° C.

### MATERIAL

Fifty normal women and 47 patients suffering from breast carcinoma were studied. A total of 368 estimations was performed on these subjects both before and after treatment. One hundred and ninety-three of these estimations were performed before the patients received treatment (apart from androgens which in many cases had been given before the patient was referred, and operative procedures such as removal of the primary lesion which in some cases had been performed many years before) and these 193 estimations form the basis of this report. It is hoped to publish the findings with regard to the effect of treatment on the enzyme levels in a later report.

1. *Normal controls.*—Fifty normal women aged from 18 to 70 years served as controls (Table I). Thirty-four of these were hospital patients about to undergo operation for hernias, varicose veins, etc. The remainder were students and members of the hospital staff. Thirty of the 50 were premenopausal and 20 menopausal or postmenopausal. The overall mean value of $\beta$-glucuronidase for these 50 individuals was 3·58 units and the standard deviation 1·31. The normal range was taken as $\pm 2\sigma$ from the mean, i.e. 0·96 to 6·20 units.

The mean values for the pre- and postmenopausal groups were almost identical being 3·57 and 3·59 respectively.

2. *Cases of breast cancer.*—The enzyme levels and relevant clinical details for the 47 cases of breast cancer are summarized in Table II. The mean $\beta$-glucuronidase for this group irrespective of staging or previous therapy is 6·90 units (standard deviation 3·87). This figure is significantly greater than the mean value of the control group ($c = 5{\cdot}63$ $P = < 0{\cdot}0000001$). Fifty-one per cent of these cases had enzyme levels within the normal range and 49 per cent had elevated readings.

### *Analysis of cases*

An analysis of these figures was undertaken in an attempt to correlate them with the clinical and pathological findings in individual cases. The results are expressed graphically in Fig. 1.

(a) *Clinical staging.*—Of the 47 cases 10 (21·25 per cent) were clinically classed as stage I or II (Table II). The mean value for this group is 6·13 units (standard deviation 4·07). A comparison of this with the control group shows the difference to be significant ($t = 3{\cdot}67$ $P = < 0{\cdot}001$).

The remaining 37 cases were classed as stage III or IV and the mean value for this group is 7·11 (standard deviation 3·84). Comparison with the control group gives a value for $c$ of 5·35 and for $P$ of $< 0{\cdot}0000001$.

Comparison of the stage III and IV group with the stage I and II group however shows a difference in means of only 0·98. This does not attain statistical significance ($t = 0{\cdot}71$ $P = < 0{\cdot}5$).

(b) *Previous hormone therapy.*—Of the 37 cases in the stage III and IV group 20 had received treatment with androgenic hormones usually methyl testosterone by mouth or testosterone propionate by injection at some time in the course of their disease.

Cases 31, 33, 34, and 38 in the androgen treated group and 32, 35, 36, and 37 in the non-treated group had clinical evidence of liver involvement at the time of the estimations and are excluded. This leaves for comparison 16 treated and 13 untreated cases. The mean values of $\beta$-glucuronidase for these two groups are 7·99 and 5·61 units respectively. With the number of cases available this does not indicate a significant difference ($t = 1{\cdot}56$, $P = < 0{\cdot}2$).

(c) *Hepatic involvement.*—Twenty-nine of the 37 cases in the stage III and IV group had no clinical or laboratory evidence of liver damage. Nine cases had either enlarged livers, jaundice or abnormal liver function tests. In one case (No. 26) observations were made both before and after the development of signs of liver metastasis and she is therefore included in both groups.

TABLE I.—*Normal Controls*

| Control number | Age | Relation to menopause | Number of readings | Mean β-glucuronidase |
|---|---|---|---|---|
| 1 | 30 | Premenopausal | 1 | 4·30 |
| 2 | 37 | ,, | 1 | 1·85 |
| 3 | 22 | ,, | 1 | 1·27 |
| 4 | 18 | ,, | 3 | 1·40 |
| 5 | 21 | ,, | 1 | 3·13 |
| 6 | 22 | ,, | 1 | 2·25 |
| 7 | 28 | ,, | 1 | 2·48 |
| 8 | 25 | ,, | 1 | 3·24 |
| 9 | 32 | ,, | 1 | 3·40 |
| 10 | 32 | ,, | 1 | 3·16 |
| 11 | 27 | ,, | 1 | 3·51 |
| 12 | 22 | ,, | 1 | 2·75 |
| 13 | 39 | ,, | 1 | 5·01 |
| 14 | 31 | ,, | 1 | 3·86 |
| 15 | 62 | Postmenopausal | 1 | 4·53 |
| 16 | 63 | ,, | 1 | 2·75 |
| 17 | 44 | Premenopausal | 1 | 2·32 |
| 18 | 55 | Postmenopausal | 1 | 1·97 |
| 19 | 52 | Menopausal | 2 | 4·87 |
| 20 | 55 | Postmenopausal | 1 | 4·60 |
| 21 | 53 | Menopausal | 1 | 2·35 |
| 22 | 63 | Postmenopausal | 1 | 3·80 |
| 23 | 65 | ,, | 1 | 3·36 |
| 24 | 40 | Premenopausal | 1 | 1·67 |
| 25 | 70 | Postmenopausal | 1 | 4·95 |
| 26 | 21 | Premenopausal | 1 | 5·00 |
| 27 | 55 | Menopausal | 1 | 2·44 |
| 28 | 21 | Premenopausal | 1 | 3·95 |
| 29 | 21 | ,, | 1 | 3·66 |
| 30 | 21 | ,, | 1 | 3·86 |
| 31 | 18 | ,, | 1 | 3·90 |
| 32 | 62 | Postmenopausal | 1 | 3·90 |
| 33 | 52 | Menopausal | 1 | 2·32 |
| 34 | 28 | Premenopausal | 1 | 4·60 |
| 35 | 54 | Postmenopausal | 1 | 6·20 |
| 36 | 57 | ,, | 1 | 3·20 |
| 37 | 29 | Premenopausal | 1 | 4·50 |
| 38 | 39 | ,, | 1 | 8·50 |
| 39 | 28 | ,, | 1 | 4·85 |
| 40 | 22 | ,, | 1 | 4·20 |
| 41 | 40 | ,, | 1 | 2·44 |
| 42 | 57 | Postmenopausal | 1 | 5·05 |
| 43 | 51 | ,, | 1 | 4·10 |
| 44 | 49 | ,, | 1 | 2·73 |
| 45 | 37 | Premenopausal | 1 | 4·00 |
| 46 | 46 | Postmenopausal | 1 | 3·86 |
| 47 | 39 | Premenopausal | 1 | 3·30 |
| 48 | 61 | Postmenopausal | 1 | 2·00 |
| 49 | 29 | Premenopausal | 1 | 4·61 |
| 50 | 46 | Postmenopausal | 1 | 2·84 |

The mean $\beta$-glucuronidase level of the group without liver secondaries was 6·93 and of the other group 7·26. This difference is not significant ($t = 0{\cdot}34$, $P = < 0{\cdot}8$). Five of the cases in the hepatic group were actually jaundiced at one stage of their disease. The mean value for this group (No. 26, 32, 34, 37 and

TABLE II.—*Breast Cancer*

| Case number | Age | Relation to menopause | Androgen therapy | Hepatic metastasis | Histology | Number of readings | Mean $\beta$-glucuronidase | |
|---|---|---|---|---|---|---|---|---|
| 1 | 80 | Post. | — | — | Spheroidal | 1 | 10·47 | |
| 2 | 48 | Men. | — | — | ,, | 1 | 14·70 | |
| 3 | 55 | Post. | — | — | ,, | 1 | 4·85 | |
| 4 | 71 | ,, | — | — | Poorly diff. | 1 | 5·91 | Stage I |
| 5 | 68 | | — | — | ,, ,, | 1 | 3·70 | |
| 6 | 59 | ,, | — | — | Mucoid. | 1 | 2·42 | |
| 7 | 71 | ,, | — | — | Scirrhous | 1 | 1·85 | |
| 8 | 48 | ,, | — | — | Spheroidal | 1 | 7·70 | |
| 9 | 34 | Pre. | — | — | ,, | 1 | 2·43 | Stage II |
| 10 | 34 | ,, | — | — | Anaplastic | 1 | 7·25 | |
| 11 | 47 | ,, | — | — | Not known | 4 | 2·55 | |
| 12 | 49 | ,, | — | — | Undiff. | 3 | 1·90 | |
| 13 | 55 | Post. | — | — | Spheroidal | 2 | 3·50 | |
| 14 | 76 | ,, | + | — | ,, | 8 | 5·87 | |
| 15 | 51 | ,, | + | — | Scirrhous | 3 | 4·84 | |
| 16 | 54 | ,, | — | — | Not known | 4 | 18·62 | |
| 17 | 35 | Pre. | + | — | Undiff. | 4 | 18·80 | |
| 18 | 52 | Men. | + | — | Scirrhous | 1 | 5·05 | |
| 19 | 42 | Pre. | + | — | Anaplastic | 1 | 9·00 | |
| 20 | 46 | Rad. men. | + | — | ,, | 6 | 7·32 | |
| 21 | 79 | Post. | — | — | ,, | 2 | 4·12 | |
| 22 | 37 | Rad. men. | + | — | ,, | 2 | 11·50 | |
| 23 | 78 | Post. | — | — | Not known | 10 | 7·52 | |
| 24 | 58 | ,, | — | — | Scirrhous | 2 | 5·27 | |
| 25 | 73 | ,, | — | — | ,, | 8 | 5·79 | |
| 26 | 32 | Pre. | + | — | ,, | 5 | 4·32 | |
| 27 | 56 | Men. | + | — | Spheroidal | 4 | 3·67 | |
| 28 | 65 | Post. | + | — | Not known | 1 | 10·25 | |
| 29 | 49 | Pre. | + | — | Spheroidal | 3 | 11·25 | |
| 30 | 54 | Post. | + | — | Anaplastic | 3 | 4·05 | Stages III and IV |
| 31 | 47 | Pre. | + | + | Not known | 5 | 6·58 | |
| 32 | 48 | Post. | — | + | ,, ,, | 2 | 4·73 | |
| 33 | 54 | ,, | + | + | Scirrhous | 10 | 7·99 | |
| 34 | 52 | ,, | + | + | Not known | 1 | 6·60 | |
| 35 | 55 | ,, | — | + | Scirrhous | 2 | 4·72 | |
| 36 | 58 | ,, | — | + | Paget's and scir. | 5 | 8·83 | |
| 37 | 53 | ,, | — | + | Not known | 3 | 12·90 | |
| 38 | 43 | Post-hypo. | + | + | Anaplastic | 5 | 9·85 | |
| 39 | 47 | Pre. | — | — | Scirrhous | 4 | 4·36 | |
| 40 | 65 | Post. | — | — | Not known | 3 | 4·57 | |
| 41 | 58 | ,, | — | — | Spheroidal scirrhous | 1 | 4·33 | |
| 42 | 52 | ,, | + | — | Mucoid | 2 | 8·80 | |
| 43 | 59 | ,, | + | — | Not known | 6 | 9·44 | |
| 44 | 45 | , | + | — | ,, ,, | 1 | 7·00 | |
| 45 | 63 | ,, | + | — | ,, ,, | 6 | 6·66 | |
| 46 | 62 | ,, | — | — | ,, ,, | 1 | 4·00 | |
| 47 | 54 | ,, | — | — | Scirrhous simplex | 2 | 6·35 | |
| 26*b* | .. | .. | + | + | .. | 2 | 6·62 | |

38) during the jaundiced period was 8·59 which of course, for so few cases is not outside the range of chance deviation.

(d) *Histology*.—The histology of the lesion was known in 34 of the 47 cases. Twenty-one of these were described as anaplastic and the remainder were scirrhous carcinomas of varying degrees of differentiation. The mean values of glucuronidase for the two groups are 7·41 and 5·16 ($t = 1{\cdot}76$, $P = < 0{\cdot}1$).

FIG. 1.—Comparison of plasma β-glucuronidase levels of 50 normal women with 47 cases of breast cancer. The dotted horizontal line represents the mean value for the control group and the upper and lower continuous lines two standard deviations around this mean.

### DISCUSSION

The difference in levels between cancerous and non-cancerous individuals in this series is highly significant. This corresponds with the findings of Goldbarg, Pineda, Banks and Rutenburg (1959), but not with those of Cohen and Huseby (1951).

The reason for this discrepancy between different workers is not clear. The method used by Cohen and Huseby (1951) is that of Tallalay, Fishman and Huggins (1946) while Goldbarg, Pineda, Banks and Rutenburg (1959) used 6-bromo-2-naphthyl β-D-glucopyruronoside as a substrate.

The method used in the present series is a modification of the method of Tallalay, Fishman and Huggins (1946) suggested by Boyland, Wallace and Williams (1955).

The estimations have been carried out on oxalated plasma without the addition of protein precipitating agents such as the trichloracetic acid advocated by Fishman, Springer and Brunetti (1948) and also used by Goldbarg, Pineda, Banks and Rutenburg (1959).

The difference in means between the stage I and II group and the stage III and IV group is not sufficiently large in relation to the number of cases to be outside the range of chance but serial observations on individual patients have frequently demonstrated a rising titre corresponding to deterioration in clinical state. Such a case is illustrated in Fig. 2.

FIG. 2.—Serial estimations of plasma β-glucuronidase (case 23) showing rise in levels with advance in disease process.

It seems likely that with larger numbers of cases this gradient will attain significant proportions.

The findings with regard to hepatic metastasis do not correspond with those of Goldbarg, Pineda, Banks and Rutenburg (1959) who found raised levels in a majority of cases of hepatic metastasis from breast cancer. Though in the 5 jaundiced cases very high levels were seen which suggest again that with a larger number of cases the difference might be significant.

The correlation with histological grading is of interest, suggesting as it does, the possibility that there may be less β-glucuronidase activity in those patients with scirrhous types of growth.

The question of hormonal effects on tumours and on β-glucuronidase is of considerable complexity and these results do not as yet allow of any but the most tentative suggestions as to their relationship. It is perhaps of interest, however, to note that the ratio of normal to abnormal levels in the cancer group (51 and 49 per cent) is not very far removed from the percentages given by Baron, Gurling and Radley-Smith (1958) and Luft, Olivecrona, Ikkos, Nilsson and Ljunggren (1956) for remissions and failures following hypophysectomy. This may of course be entirely coincidental but investigations are in progress to ascertain whether any relationship exists.

SUMMARY

(1) An investigation into the plasma $\beta$-glucuronidase levels of 47 cases of breast cancer and 50 normal women has been undertaken using a modification of the method of Tallalay, Fishman and Huggins (1946).

(2) The difference between the enzyme levels of the cancerous and non-cancerous groups was highly significant.

(3) Stage I and II carcinomas were associated with significantly raised enzyme levels. Stage III and IV cases had higher mean values than Stage I and II but the difference was within the range of chance.

(4) Cases who had received androgen therapy had somewhat higher mean values than those who had not had androgens and those with anaplastic growths had higher values than those with scirrhous but chance variation cannot be excluded as a cause for this.

(5) No great difference in enzyme levels could be demonstrated between cases with and without hepatic metastasis in the absence of jaundice.

I wish to express my thanks to Mr. E. J. Radley-Smith and Dr. D. N. Baron for sponsoring this work and for their advice and encouragement ; also to Dr. D. C. Williams whose very considerable experience in this field has been of the greatest help in overcoming technical and other difficulties ; to the Consultant Staff of the Royal Free Hospital who have generously allowed me access to their patients ; and to Sister Hitchcock without whose help and co-operation the collections of specimens would have been impossible.

Financial support for the apparatus and material used in the investigation was generously provided by the British Empire Cancer Campaign.

### REFERENCES

Baron, D. N., Gurling, K. J. and Radley-Smith, E. J.—(1958) *Brit. J. Surg.* **45**, 593.

Boyland, E., Gasson, J. E. and Williams, D. C.—(1957) *Brit. J. Cancer*, **11**, 120.

*Idem*, Wallace, D. M. and Williams, D. C.—(1955) *Ibid.*, **9**, 62.

Cohen, S. L. and Huseby, R. A.—(1951) *Proc. Soc. exp. Biol. N.Y.*, **76**, 304.

Fishman, W. H., Kasdon, S. C., Bonner, C. D., Fishman, L. W. and Homburger, F.—(1951) *J. clin. Endocrin.*, **11**, 1425.

*Idem*, Springer, B. and Brunetti, R.—(1948) *J. biol. Chem.*, **173**, 449.

Goldbarg, J. A., Pineda, E. D., Banks, B. M. and Rutenburg, A. M.—(1959) *Gastroenterology*, **36**, 193.

Kerr, L. M. H. and Levvy, G. A.—(1947) *Nature, Lond.*, **160**, 463.

Levvy, G. H., Kerr, L. M. H. and Campbell, J. G.—(1948) *Biochem. J.*, **42**, 462.

Luft, R., Olivecrona, H., Ikkos, D., Nilsson, L. and Ljunggren, H.—(1956) *Amer. J. Med.*, **21**, 728.

Tallalay, F., Fishman, W. H. and Huggins, C.—(1946) *J. biol. Chem.*, **166**, 757.